# Neutron Insights into Sorption Enhanced Methanol Catalysis

**DOI:** 10.1007/s11244-021-01461-w

**Published:** 2021-06-06

**Authors:** Marin Nikolic, Luke Daemen, Anibal J. Ramirez-Cuesta, Rafael Balderas Xicohtencatl, Yongqiang Cheng, Seth T. Putnam, Nicholas P. Stadie, Xiaochun Liu, Jasmin Terreni, Andreas Borgschulte

**Affiliations:** 1 Laboratory for Advanced Analytical Technologies, Empa - Swiss Federal Laboratories for Material Science and Technology, Überlandstrasse 129, 8600 Dübendorf, Switzerland; 2 Department of Chemistry, University of Zurich, Winterthurerstrasse 190, 8057 Zurich, Switzerland; 3 Oak Ridge National Laboratory, Neutron Spectroscopy Division, Spallation Neutron Source (SNS), Oak Ridge, TN 37831-6475 USA; 4 Department of Chemistry and Biochemistry, Montana State University, Bozeman, MT 59717-3400 USA

**Keywords:** Sorption enhanced catalysis, Inelastic neutron scattering, CO_2_ hydrogenation, Methanol, Dimethyl ether

## Abstract

Sorption enhanced methanol production makes use of the equilibrium shift of the $\hbox {CO}_2$ hydrogenation reaction towards the desired products. However, the increased complexity of the catalyst system leads to additional reactions and thus side products such as dimethyl ether, and complicates the analysis of the reaction mechanism. On the other hand, the unusually high concentration of intermediates and products in the sorbent facilitates the use of inelastic neutron scattering (INS) spectroscopy. Despite being a post-mortem method, the INS data revealed the change of the reaction path during sorption catalysis. Concretely, the experiments indicate that the varying water partial pressure due to the adsorption saturation of the zeolite sorbent influences the progress of the reaction steps in which water is involved. Experiments with model catalysts support the INS findings.

## Introduction

**Fig. 1:**
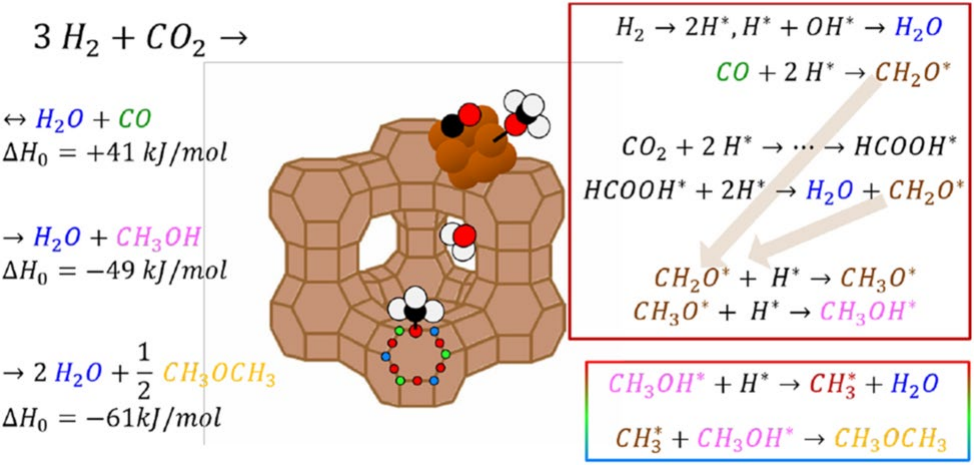
On the left side, the overall reaction formulas leading to the observed [7] products from $\hbox {CO}_2$ reduction on Cu impregnated 13× zeolites are given, while the right side emphasizes potential intermediates of $\hbox {CO}_2$ hydrogenation on copper calculated by Grabow and Mavrikakis [10]. The color code for the compounds CO, methanol, and dimethylether is retained throughout all figures. In contrast to typical catalysis, the amount of products and intermediates ‘stored’ on the support (sorbent) is significantly higher (sketch). Formation of CO and methanol are assumed to take place on the copper surface (brown box), while dimethyl ether is formed on the zeolite [11] (coloured box). In both cases, methoxy groups are crucial, although the bonding strengths of O to Cu and O to Si are different [12]

Methanol as a renewable fuel can be produced by reduction of $\hbox {CO}_2$ with hydrogen over $\hbox {Cu/ZnO/Al}_2\hbox {O}_3$ catalysts [1–3]. The high thermodynamic stability of $\hbox {CO}_2$ results in a relatively low driving force of the reaction [4]. In addition, the kinetics are slow due to limited transport/desorption of products from the active centers and thus the overall reaction yield is low [5]. The thermodynamics can be positively influenced by (Le Chatelier principle), and kinetic constraints can be lowered by the use of so-called sorption enhanced catalysis [4, 6–8]. The concept of sorption enhanced catalysis makes use of the fact that the reaction kinetics are controlled by the concentration of reactants and products at the reaction centres, which is modified by active removal of the product. This may be achieved with the help of selective membranes or ionic liquids [9]. Alternatively, the support of the catalyst is replaced by a material being able to adsorb the product(s) to a large extent, e.g., by a zeolite [4, 6]. Terreni et al. [7] demonstrated an improvement of the reaction yield of more than a factor of two by sorption enhancement (Fig. 1) compared to the steady state reaction, but at an overall low rate. Further improvement needs the identification of the rate limiting steps in the complex reaction. In particular, although not intended, the zeolite sorbent catalyzes the formation of dimethyl ether from methanol increasing the complexity of the overall reaction.

**Fig. 2:**
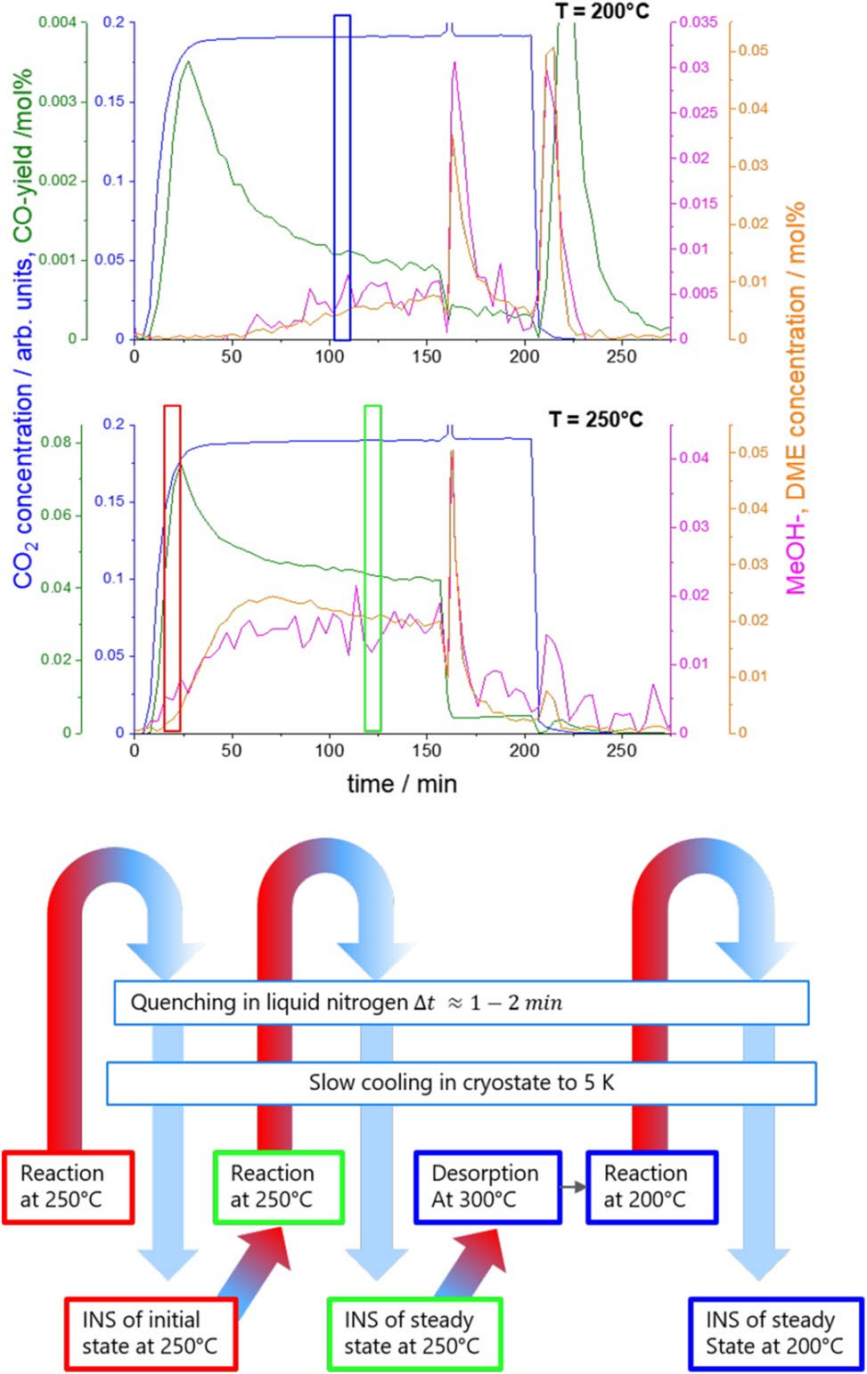
Upper panel: CO, methanol and dimethyl ether yields from hydrogenation of $\hbox {CO}_2$ over a Cu zeolite catalyst at 15 bar and 200 $^\circ$C and 250 $^\circ$C, respectively. The total pressure is lowered to 1 bar at $t=160$ min, and subsequently ($t=210$ min) the sample is heated. Most of the products accumulate in the sorption catalyst, as detected by the subsequent release upon pressure release and temperature increase (figures taken from Ref. [7]). The plug flow reactor setup allowed the measurement of the produced gases. For INS measurements, the reaction took place in a batch reactor with as similar conditions as possible but without product analysis. The boxes indicate the times, at which the sample (the reactor) was quenched in liquid nitrogen and further cooled down in the cryostate down to 5 K, where it is analyzed by INS (Fig. 3)

Diffusive reflectance Fourier transform spectroscopy (DRIFTS) is a standard operando method to access intermediates on catalysts [13–15]. However, the high pressure needed impedes the analysis of the reaction mechanism by operando DRIFTS. This is not a general challenge, but for the methanol case the signal from high gas density of reactants ($\hbox {CO}_2$) and products (CO) drown out the weak signal from the catalyst surface [1]. Recently, the general applicability of inelastic neutron scattering (INS) as a post-mortem analysis method revealing insights into the reaction mechanism of methanol synthesis has been demonstrated by Kandemir et al. [16]. Inelastic neutron scattering is a powerful technique to study vibrational properties of materials [17]. INS spectra may be very similarly interpreted as those derived by DRIFTS [18]. The scattering process is not influenced by optical selection rules, and neutrons have a particular high cross section towards hydrogen atoms [17]. The INS spectra of the reaction are thus less affected from $\hbox {CO}_2$ and CO, and reveal quantitative information of the hydrogen containing products (Fig. 1). However, INS is performed at low temperature (T < 20 K) to reduce the large Debye–Waller factor from hydrogen, and thus a post-mortem method. The use of post-mortem methods for catalysis is debated, though, because it is unknown, whether the chemical state of the reaction is unchanged during cooling (quenching, see Fig. 2). For the system under study, we benefit from the circumstance that reaction and desorption of species in sorption catalysts is kinetically constrained. The concentration of hydrogen containing compounds is high enough due to the high (hydrogen) pressure, relatively low reaction temperatures and the high surface area of the sorption catalyst, to allow the use of inelastic neutron scattering as a virtual operando spectroscopy.

## Experimental Section

### Preparation and Characterization of the Catalyst

#### Preparation

Copper as a catalytic entity was supported on commercial 13× zeolite beads (ZEOCHEM) by wet impregnation/ion exchange from a 3 M solution of $\hbox {Cu(NO}_3)_2 \cdot ~6~\hbox {H}_2\hbox {O}$ in water. After 1 week at room temperature, the beads were removed from the solution and rinsed three times with deionized water (3 $\times$ 20 mL). After drying the zeolites in a muffle oven for 64 h at 125 $^\circ$C, the zeolites were reduced in a plug flow reactor for 4 h at 400 $^\circ$C in a flow of hydrogen.

#### Catalysis

The catalytic measurements were performed using a magnetic suspension balance (Rubotherm, Bochum, Germany) equipped with an IR gas analyzer. The FTIR spectrometer (Bruker Alpha) allowed the detection of the following gases: CO, $\hbox {CO}_2$, $\hbox {CH}_3\hbox {OH}$, $\hbox {CH}_3\hbox {OCH}_3$, and $\hbox {H}_2\hbox {O}$ (Fig. 2). CO, $\hbox {CH}_3\hbox {OH}$, and $\hbox {CH}_3\hbox {OCH}_3$ were quantitatively analyzed. The results were published with more detail in [7]. In short: typical catalysis experiments neglect the effects taking place before steady-state of the gas–solid interaction has been achieved [6]. However, in sorption catalysis, the transient response is the sought effect. An experiment starts with a fully desorbed sorption catalyst. The local concentrations of reactants and products depend on the ability of the sorbent to adsorb them, which are thereby removed from the catalytically active sites. After some time, the sorbent is saturated, and now the product yield reaches steady-state. Product yield by gas analysis includes only the transient yield, which is the steady state yield after equilibration. The sorption enhanced yield must include the transient yield at initial state, plus the accumulated amount of products in the sorbent. To quantify them, the pressure of the system is first lowered (‘pressure desorption’), and then additionally heated (‘temperature desorption’), and the thereby desorbed gases analyzed [7]. Figure 4 condenses the outcome of such an experiment by comparing the space-time yields.

The same measurement procedures were performed on macroscopic 1:1 mixtures of a commercial $\hbox {Cu/ZnO/Al}_2\hbox {O}_3$ catalyst with zeolite 5 Åand zeolite 3 Å, respectively, under identical experimental conditions. The characterisation of the commercial catalyst used (Alfa Aesar, Germany) was published in [1]; the zeolites pellets of mm size were purchased from Sigma Aldrich. For discussion, we show only the space-time yields of the steady-state and at maximum rates including sorption enhancement (Fig. 4) following the procedure as described in [7].

#### Characterization

Specific surface area was determined by measuring nitrogen adsorption at 77 K between 0.05 and 800 mbar using an automated volumetric instrument (3Flex, Micromeritics Instrument Corp.) after degassing under oil-free vacuum at 200 $^\circ$C down to $10^{-6}$ mbar for 12 h. The surface area of Cu13X, fitted to the Brunauer–Emmett–Teller (BET) model between $P/P_0 = 10^{-4}$ to $P/P_0 =0.13$, was determined to be 14 m$^2$ g^−1^. The crystal structure was analyzed by X-ray diffraction (XRD, PANalytical X‘Pert Pro). The XRD patterns of pristine zeolite and the Cu-sorption catalyst indicate a partial degradation of the zeolite accompanied with the formation of an amorphous phase, which is in agreement with the observed BET-surface area decrease by a factor of four, probably caused by hydrothermal decomposition and/or impregnated/incorporated copper particles that block the pores. For further details, we refer to [7].

### Inelastic Neutron Scattering

**Fig. 3:**
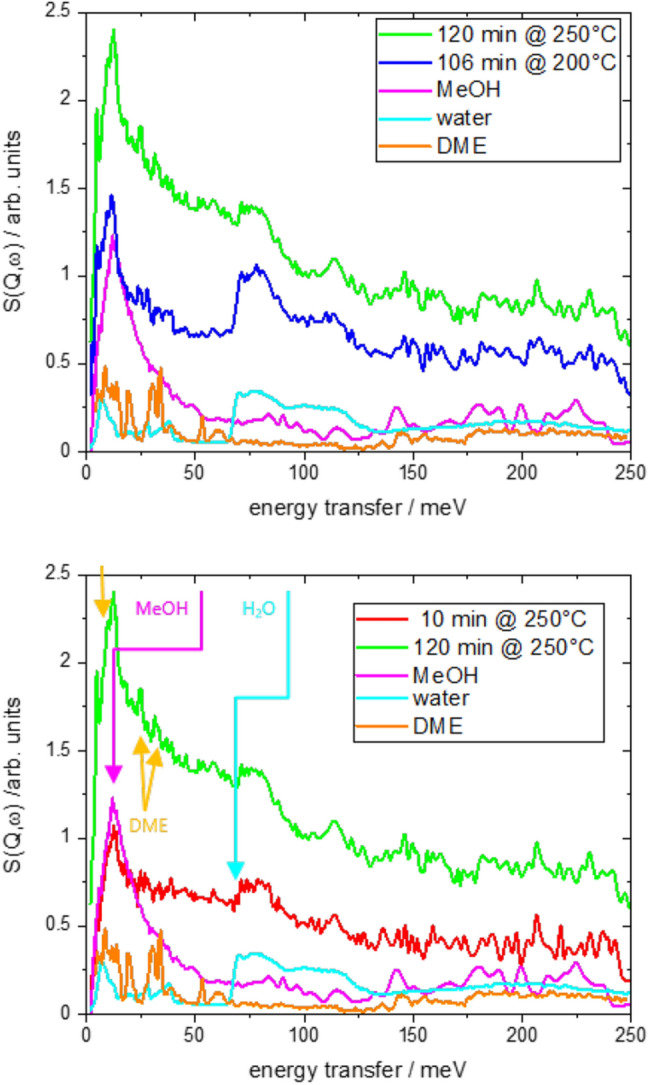
INS of Cu–Zeolite exposed to H$_2$ and $\hbox {CO}_2$ at various conditions. Upper panel shows the INS data at the same pressure (16 bar), different reaction temperatures of 250 $^\circ$C and 200 $^\circ$C, respectively, and quenched at similar reaction times (120 min and 106 min, respectively). The panel below compares the INS spectra obtained at 250 $^\circ$C at different reaction times. In all cases, the INS spectrum of methanol adsorbed on the zeolite, dimethyl ether on alumina (from Ref. [19]) and water are added for comparison

High-resolution INS spectra were measured at BL16-B (VISION), SNS, ORNL. Before measuring, the sample was heated in vacuum at 300 degree C to remove water and contamination from air. After collecting the INS data for 12 h on the clean sample, the samples were taken out of the cryostat. To follow the reaction, a precise reaction/quenching/measurement protocol is followed, which is described in Fig. 2. A stainless steel was used both for reaction as well as INS measurement without removing the sample from it. The neutron background generated by the cell was measured before the cycle, and subtracted from each later spectra. For comparison, 1.81 g methanol was dosed on Cu–zeolite 13$\times$ and measured by INS. The specific amount of methanol was chosen by an estimate of the catalytically produced amount. Indeed, the absolute signal intensity of methanol and reaction products match rather well (Fig. 3).

## Results and Discussions

Unlike most catalytic processes, substantial amounts of the reactants, intermediates, and products accumulate in the sorption catalyst. The corresponding amount may be traced back from an analysis of the product gas (e.g., from Fig. 2). This is straightforward in simple reactions, in which only one species is adsorbed. This is the case, e.g., for the sorption enhanced methanation reaction, in which only water is adsorbed, while methane leaves the sorption catalyst [6]. Obviously, this is not the case for the $\hbox {CO}_2$ reduction to methanol and further to dimethyl ether. From the product analysis in Fig. 2, one extracts that CO is hardly adsorbed, but $\hbox {CO}_2$, methanol and dimethyl ether in addition to water. Furthermore, the complicated time evolution of the gas concentrations indicate that also the concentration of the species in the sorption catalyst varies with time.

DRIFTS measurements, although truly operando, revealed only qualitative data. This difficulty can partly be overcome using inelastic neutron scattering. The neutron scattering intensity originates mainly from hydrogen, and is within one experiment directly related to the number of protons. With the spectroscopic signature we can thus probe the various species in the sorption catalyst quantitatively.

Figure 3 shows the INS-data of Cu/zeolite catalyst post-mortem quenched after a reaction time as defined in Fig. 2. Let us first discuss the spectrum at $T=$ 200 degree C and $T=$ 250 $^\circ$C after long reaction (top of Fig. 3, for a precise definition of experimental details we refer to Fig. 2). Both curves look very similar being basically a superposition of methanol, dimethyl ether and water. The latter is clearly visible by the step-like function at around 66 meV, while the rather featureless methanol spectrum (typical recoil spectrum [19]) is indicated by the maximum at around 20 meV. The various peaks are assigned to dimethyl ether (Fig. 3). The main difference between the two curves is the relative amount of the three compounds, with water dominating the 200 $^\circ$C spectrum.

Aim of this paper is the application of INS to yield information on the reaction mechanism. For this, we quenched the reactor after two different reaction times, while keeping all other parameters. The two spectra are strikingly similar. As expected from Fig. 2, the amounts of methanol and dimethyl ether increase with reaction time, which is in perfect agreement with the INS measurements. What is surprising, though, is that the ‘water edge‘ a around 66 meV remains constant. This means that although the methanol and dimethyl ether yields increase, the by-product water does not increase. This is only possible if one considers an involvement of CO in the reaction to methanol. CO, although not directly visible by INS but indirectly by the occurrence of water, is first produced due to enhanced water sorption. The preference of CO over methanol is most likely due to kinetic reasons. As sketched in Fig. 1, CO formation proceeds via two steps (adsorption and dissociation) [1, 10], while several more are needed to produce methanol and subsequently dimethyl ether. Thermodynamically, the reversed gas–water shift reaction yielding CO is endothermic and becomes exothermic with simultaneous water sorption. However, with filling up the sorbent, this effect becomes less pronounced, and the thermodynamically more favorable methanol and dimethyl ether reactions take over. The INS data also explain a small detail, which is visible in Fig. 3: the dimethyl ether yield first lacks behind that of methanol, but increases faster before it slowly decreases again. Clearly, formation of dimethyl ether is preferred even over methanol, if water sorption is possible. That dimethyl ether formation is possible, although at a lower yield at later stages of the reaction, indicates that water independent reaction steps take place, because the water signal (amount of water adsorbed in the zeolite) does not increase between the two spectra (Fig. 3).

Grabow and Mavrikakis calculated the reaction mechanisms of both CO and $\hbox {CO}_2$ to methanol and indicated various crossing points between the reactions [10]. CO reacts with hydrogen to $\hbox {CH}_2\hbox {O}^*$, an intermediate from which eventually methanol is formed (see Fig. 1, and details in Ref. [10]). The reaction path starting from $\hbox {CO}_2$ proceeds over $\hbox {CH}_2\hbox {O}^*$ as well, however, there are several highly activated reaction steps before reaching this intermediate. Furthermore, the reaction step of CO to $\hbox {CH}_2\hbox {O}^*$ proceeds without the formation of water, in contrast to the $\hbox {CO}_2$ pathway. This step is thus unaffected by the water partial pressure, which increases with filling of the sorbent. Furthermore, all water releasing steps on the way to methanol are the initial reaction steps.

Similar, the formation of dimethyl ether proceeds via the reaction of methanol with acidic hydroxyl groups bonded to Si in the zeolite lattice [11]. This yields water and a methyl group, which further reacts with methanol to dimethyl ether (see Fig. 1). The methyl groups may be formed already at early stages, explaining the formation of dimethyl ether without additional water formation.

Both effects, ‘late’ methanol and dimethyl ether formation indicate that a substantial amount of intermediates (CO, methoxy, and methyl groups) are formed at early stages and accumulate in the sorption catalyst. As the final reaction steps are now rate limiting, the water signal is comparably higher at lower temperature (Fig. 3), because the zeolite can adsorb more water at lower temperatures, while formation of methanol/dimethyl ether is slow. This is in good agreement with the fact that the overall space-time yield of the sorption catalyst is generally rather low, when compared to optimized Cu/ZnO catalysts. Terreni et al. [7] account the low yield with the large copper particles leading eventually to a comparably low number of active (Cu-) sites. There is ongoing work to improve the copper particle size.

**Fig. 4:**
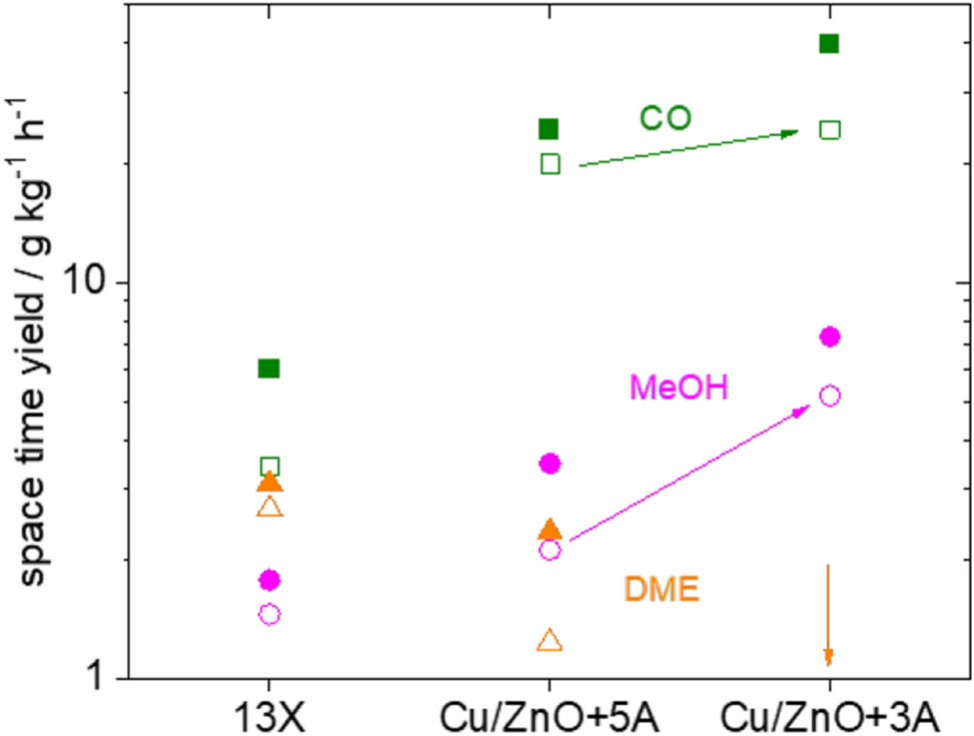
Space-time yields of the Cu–zeolite catalyst (13X) and macroscopic mixtures of a commercial $\hbox {Cu/ZnO/Al}_2\hbox {O}_3$ catalyst with zeolite 5A and zeolite 3A, respectively. The full symbols represent the maximum yields including sorption enhancement, the empty symbols are the steady-state yields. Formation of dimethylether was not detected on $\hbox {Cu/ZnO/Al}_2\hbox {O}_3+3\hbox {A}$ as indicated by arrow

This spectroscopic work, however, indicates of a direct interplay between catalysis on (Cu-) metal sites and acidic sites of the zeolites. This opens the possibility of new opportunities in sorption enhanced catalysis. Unfortunately, the number of experimental parameters multiplies with introducing additional components, in particular if having different functionalities. The experiments summarized in Fig. 4 are a simplified way to proof some of the statements extrapolated from the INS measurements using model catalyst. The zeolites added to the commercial $\hbox {Cu/ZnO/Al}_2\hbox {O}_3$ catalyst are very similar, the main difference is the pore size of 3 Å and 5 Å, respectively. However, even in the steady-state the yields differ markedly. The DME yield is practically zero for the 3 Åmixture, as methanol cannot enter the pores of the zeolite 3 Å  [20]. Water, though, is adsorbed by both zeolites [20]. The methanol yield is apparently higher in the mixture with zeolite 3 Å than in the one with 5 Å. However, considering methanol and its precursor molecules as precursors for DME, the activity of the $\hbox {Cu/ZnO/Al}_2\hbox {O}_3$ catalyst is very similar: two molecules of methanol form one dimethylether. On the other hand, also the CO yield is slightly higher for 3 Å, in particular with sorption enhancement. This proofs that catalyst and sorbent communicate over distances as large as mm lengths. This means that despite being independent reactions, methanol and dimethylether formation are influenced by each other‘s due to the different concentrations of products of the one reaction being the reactant, intermediate or inhibitor of the other. In sorption catalysts, this interaction is not restricted to volatile intermediates as is the case in macroscopic mixtures. Competitive adsorption and new (diffusion) path ways as indicated by INS may open new ways of catalysis [21].

## Conclusions

State-of-the-art sorption catalysts for methanol under-perform in terms of turn over frequency. Further catalyst developments require a better understanding of the reaction mechanism. Inelastic neutron scattering delivered quantitative information on the product yields of methanol, dimethyl ether and water during initial and steady-state of the reaction. The formed water is initially removed from the reactive sites to the zeolite, until it is saturated. Thereby, the water partial pressure varies over the course of the reaction influencing the individual reaction steps. It was found that methanol and dimethyl ether was formed relatively late without significant water formation indicating that a substantial amount of intermediates (CO, methoxy, and methyl groups) is forming at early stages, accumulating in the sorption catalyst before finally reacting to the end product.

The beauty of the study is that despite the difficulty of the measurement procedure and complex outcome (INS spectra, e.g., of methanol without striking peaks), the interpretation of the results is straightforward, i.e., based on a simple fingerprint method. This is partly due to the fact that the INS signal is an absolute measure. Unlike DRIFTS, which depends on details of optical scattering [14, 15, 22, 23] thereby depending on optical reflectivity, sample shape, microstructure etc. The study is thus a showcase for the use of INS in heterogeneous catalysis.
